# Associations between motorized transport access, out-of-home activities, and life-space mobility in older adults in Japan

**DOI:** 10.1186/s12889-022-13033-y

**Published:** 2022-04-07

**Authors:** Yen Tran, Naohisa Hashimoto, Takafumi Ando, Toshihisa Sato, Naoki Konishi, Yuji Takeda, Motoyuki Akamatsu

**Affiliations:** The National Institute of Advanced Industrial Science and Technology (AIST), Tsukuba, Japan

**Keywords:** Life-space mobility, Transport access, Out-of-home activities, Japan

## Abstract

**Background:**

Motorized transport access and out-of-home activities are two potential correlates of Life-space mobility (LSM), a common research topic in mobility studies of older adults. These correlates remain mostly unexplored in previous literature and relating them with LSM can reveal directions for improving the LSM of older adults.

**Methods:**

The associations between motorized transport access, out-of-home activities, and LSM were examined using data from 1,333 older adults (mean age = 70.63) living in 15 cities and towns in Japan. LSM was assessed using composite life-space assessment (LSA) scores. Motorized transport access was measured using dummies showing whether a person had car access (divided into five levels) and used public transport (bus and railway), and out-of-home activities were measured using the number of various activities that were conducted during the most recent weekday and weekend day. Generalized linear models were used to assess the associations.

**Results:**

The sample was dominated by males (74.42%), with more than half of the sample had their own cars. On average, each respondent had four activities during two survey days, and shopping was the most common activity. The results showed that owning a car and using railway, as well as various activities were associated with increased composite LSA scores, whereas no cars or only shared cars in home were associated with decreased composite LSA scores. However, these associations differed between males and females.

**Conclusions:**

In this study, different levels of motorized transport access and different types of out-of-home activities were found to associate differently with composite LSA scores. Based on these findings, we suggest that policymakers should provide more transport access, pay more attention to the LSM of older adults with high clinics/hospital activities, and trigger more shopping and daily leisure activities for older adults to improve the LSM of this population.

## Background

Life-space mobility (LSM) [1] has become a common research topic in mobility studies of older adults. Unlike conventional mobility measures, LSM describes a full continuum of mobility in the daily life-space of a person, reflecting both physical conditions and environmental factors in measuring mobility. This metric has been suggested for geriatric research studies and clinical practice [2], as well as for policy interventions aimed at improving mobility [1, 3]. Empirical studies have shown that this measure can predict various health-related issues, such as physical/cognitive functions, health care utilization, depression, falls, and mortality [2–4]. Given the importance of mobility to health [5–7] and well-being [8–10] which has been strongly established in previous literature, maximizing LSM can lead to additional benefits due to spatially expanded mobility.

Theoretically, transport access (referred to the opportunity to use car or public transport [11]) can affect one’s LSM. At certain travel distances, people are generally reliant on motorized modes such as cars, buses, or railways. The lack of access to these modes can thus limit LSM. Moreover, many older adults cannot drive because of the decline in a number of physical and cognitive functions [12], or medical conditions [13, 14]. Similarly, older adults living in rural areas with poor public transport systems may have difficulty traveling. In these cases, the unavailability of motorized modes is likely to result in a limited LSM. Therefore, accesses to motorized modes can be a critical factor for one’s LSM. We, however, found no previous studies that explicitly explored this factor, although some related studies are available. For example, LSM was found to be associated with travel mode uses [15, 16], having a driver’s license [17], and transportation difficulty [3]. However, these measures do not fully reflect a person’s *opportunity* to use a certain mode. For example, using one’s own car and using a shared car might be different in terms of opportunity to use a car whenever one wants to go. Similarly, a driver’s license only indicates one’s driving ability, and it does not necessarily imply one’s actual access to car use.

Out-of-home activities are another potential factor that affects LSM. The number of out-of-home activities as well as which specific activities that people perform daily can affect LSM significantly, as different activities can result in different travel distances and destinations. Intuitively, more out-of-home activities can lead to greater LSM, as people might travel more frequently. However, uncertainty remains regarding how different types of activities affect LSM. Despite this, we found no previous studies that explored the association between out-of-home activities and LSM in older adults.

This study aims to address the two aforementioned research gaps by relating transport access and (performed) out-of-home activities with LSM. Transport access indicates how transportation is enabled, and out-of-home activities are commonly known as the purpose of transportation, e.g., people travel not for the sake of traveling itself, but rather to perform a variety of activities [18]. Examining these measures can enhance our understandings on the relationship between transportation and LSM. First, the association between motorized transport access (car, bus, and railway) and LSM is considered. More specifically, we posit the following hypothesis:

### H1: Different levels of motorized transport access correlate differently with LSM

This hypothesis considers the role of motorized transport access in LSM. Although non-motorized modes, such as walking and cycling, are necessary for older adults, particularly for short-distance trips, our focus in this study was motorized modes. Motorized modes are common objects of transport policy intervention because they often require large investments, and they can have a significant impact on society. The fact is that motorized modes affect LSM only over long distances. We however argue that ensuring adequate LSM for long-distance trips is important in many cases. In rural and distant areas in Japan, facilities necessary for daily life such as supermarkets, clinics, etc., are often sparsely distributed, which result in long travel distances. Even in urban areas, shopping facilities may be within walkable distances, but certain clinics (e.g., those for infrequent treatments) or hospitals may not exist near one’s home. In these cases, larger LSM over long distances are indicative of greater the level of autonomy that one possesses for reaching far destinations. In other countries, particularly in well-serviced (urban) areas, this issue is irrelevant because daily services can be reached within short distances. However, such cases are not common, and a large portion of the world population still lives in rural areas [19, 20] where people might have to travel long distances for daily services.

The second research gap between out-of-home activities and LSM is addressed by testing the following hypothesis:

### H2: Different out-of-home activities correlate differently with LSM

Performed out-of-home activities are both shaped by the built environment (e.g., the density of shopping centers) and directly driven by personal needs. The latter one, i.e., needs for out-of-home activities, can be considered a form of personal characteristic because these needs can sometimes be unique to a person and cannot be fully explained by common socio-demographic characteristics such as age and gender. Thus, the relationship between out-of-home activities and LSM may add value to our knowledge of how personal characteristics relate to LSM, which can be helpful for policy considerations.

In summary, our study explores the associations between motorized transport access, out-of-home activities, and LSM in older adults.

## Methods

### Data collection

The data used for this study were provided by the national Smart Mobility Challenge project in Japan, which was jointly conducted by the Ministry of Land, Infrastructure, Transport, and Tourism and the Ministry of Economy, Trade, and Industry (METI) [21]. This project was an effort to improve mobility in the country in response to urgent problems related to aging and revitalization in specific regions. At the time of writing, the project is entering its second fiscal year, and 16 cities/towns have been selected as experimental regions by METI. Improving the LSM of older adults was specified as a main objective of this project. Within this project, a web-based survey was conducted for the people living in 15 of the 16 examined regions. The survey asked the respondents to report their LSM, out-of-home activities, and the travel mode used for each of these activities in a weekday and a weekend day (the most recent ones).

The web-based survey was conducted from January 23, 2021, until February 1, 2021, by a third-party company. The survey procedure and all applied rules followed the “Act on the Protection of Personal Information,” JIS Q 15,001 (Personal Information Protection Management Systems). Invitations to join the survey were sent randomly to all residents in the 15 study regions who registered their profiles in the database of the survey company. Each respondent answered 51 questions, and their responses were recorded only when all the required questions were answered. The website for the survey was closed when the number of recorded answers reached a pre-determined number of 13,000.

For this study, we retained the responses of only respondents aged 65 and over from the total number of 13,000 responses with a known gender, who were not infected with COVID-19, and who were able to achieve at least the second level described in [1] (i.e., being able to go outside of their home with or without any assistance). This screening step was applied to explicitly exclude older adults who were not able to travel outside of their homes. The full sample thus consisted of 1,333 older adults (mean age = 70.63). In addition, potentially due to the characteristics of an online survey, the sample was strongly gender-unbalanced as the number of males (*N* = 992) was nearly three times higher than that of females (*N* = 341). To account for this imbalance, we first analyzed the associations separately for the male sample (*N* = 992) and female sample (*N* = 341). We then randomized the full sample (*N* = 1,333) to generate a gender-balanced sample that matched the gender distributions in the populations of the 15 regions. The full sample was strongly skewed towards males (i.e., the male/female ratio was 2.91), whereas the populations in all 15 regions were slightly skewed towards females (i.e., the male/female ratio was 0.78). Therefore, the randomization process only randomly selected a given number of males in a region (calculated according to the gender distributions in the regional population) from the total number of males in that region, while the number of females in that region was kept constant. This was achieved using the function “Random sample of cases” in SPSS for Windows (IBM SPSS Statistics for Windows, Version 27.0.1.0), which can randomly select a specific number of cases from a given total number of cases. The adjusted sample (*N* = 610) therefore included the 341 original female and the 269 adjusted male population, and this sample was used to draw joint conclusions concerning the investigated LSM associations for males and females.

## Measurements

### Life-space mobility

LSM was measured according to the University of Alabama at Birmingham (UAB) Study of Aging Life-Space Assessment [1], or ‘UAB Study of Aging LSA’ in short, which is the most frequently used method for determining LSM [4]. This scale was translated into Japanese by the Physical Therapy Association [22], Japan, and was used in our study. The Japanese version of this scale was also validated with a sample of 2,147 older people aged 65 and above in Japan and showed expected correlations with several conventional mobility scales (e.g., Time up and go test and Instrumental activities of daily living) [22]. In [1], travel ranges are classified into six levels ranging from Level 0, Level 1, … to Level 5. The lowest level, i.e., Level 0, is assigned if a person’s movements were limited to within his/her own bedroom. The remaining five levels are assigned if the person has moved to places other than the bedroom, such as to other rooms within his/her home (Level 1), to outside his/her home (Level 2), to his/her neighborhood (Level 3), to his/her town/city (Level 4), and to beyond his/her town/city (Level 5). At each level, the respondents were asked whether they reached that level in the last four weeks, followed by questions of how frequently they visited that level (1 = less than once a week, 2 = 1–3 times a week, 3 = 4–6 times a week, and 4 = daily) and whether they needed equipment or a person to assist in their moving to that level. Besides the original items in [1], we added a question asking the respondents to report the travel modes they used to reach each of the aforementioned levels. All possible travel modes in each region were shown for the respondents for selection (i.e., multiple selections were possible). The list of available travel modes generally included car use (self-driving, being driven, taxi, or shared car), bus use, railway use (the subway is unavailable in the examined regions), walking (alone, with a stick, with a walking frame, etc.), wheelchair, bicycle, and motorbike, and each list varied with the region. The respondents could also write the names of the travel modes they used that were not present in the listed options.

Following the instructions in [1], we first calculated a composite LSA score for each respondent with a value ranging from zero to 120 (a higher score implies a greater LSM). Each score was calculated by multiplying three numbers, including a number that represents the life-space level (‘0’ for Level 0, ‘1’ for Level 1, … ‘5’ for Level 5), a number that represents the degree of independence (‘2’ if the person did not need any equipment assistance or person assistance, ‘1.5’ if only equipment assistance was reported, and ‘1’ if person assistance was reported), and a number that represents the frequency of attainment (i.e., ‘1’, ‘2’, ‘3’, and ‘4’ as defined above). We also created a restricted life-space dummy for each respondent that utilizes a value of “yes” if one’s *independent life-space* is 3 (i.e., within one’s neighborhood) or less, or “no” otherwise. Independent life-space, or ‘LS-I’ in [1], represents the maximum life-space level that a person attained during the last four weeks without requiring any equipment/person assistance. As such, it can take as many as values that life-space levels can take, for example ‘0’ if the maximum life-space level is Level 0, ‘1’ if the maximum life-space level is Level 1, … etc. Whereas a composite LSA score provides a detailed assessment of one’s LSM, the restricted life-space dummy indicates whether one’s LSM is restricted. In this study, the composite LSA scores of the respondents were treated as a continuous variable in the regression models, and the restricted life-space dummies were used only in the descriptive summary of the samples.

### Motorized transport access

In addition to the questions regarding the LSA described above, we first asked the respondents to report whether they possess a driver’s license or not. This constitutes a measure of driving ability. Next, the respondents were asked to report the car availabilities in their homes with three answers to select, including ‘There’s one car in my home that is almost used by me only’, ‘There are shared cars in my home’, and ‘No car in home’. From these two questions, we constructed a measure of car access with five possible levels arranged in increasing levels of car access, including:

- No cars in home (Level 0);
- Shared cars only, AND:
  ∘  The respondent does NOT have a driver’s license (Level 1);
  ∘  The respondent HAS a driver’s license (Level 2);
- Owning a car only (Level 3);
- Both owning a car and sharing cars (Level 4).

Note: Level 1 implies that the respondent is driven by the family members and Level 4 implies access to both one’s own car and the shared cars in home.

Measuring public transport access was not a straightforward step in the survey because we could not access to the home addresses of the respondents due to the privacy restrictions. To overcome this problem, a proxy of public transport access was used. Specifically, the variable ‘public transport used’ was created to indicate whether the respondents had used a public transport mode (bus or railway) or not when: (1) They traveled to Level 3, 4, and 5 described in the life-space mobility measurements and when; (2) They traveled for out-of-home activities in one weekday and one weekend day. An example of relating transportation used with LSM can be found in [16]. It should be noted that while public transport use is considered as a proxy for public transport access, there are some cases this measure fails to capture its target, for example when people have public transport access (e.g., living near a bus stop) but do not use it.

Finally, the above five car access variables (i.e., car accesses of Level 0 to Level 4) and two variables for public transport used (i.e., bus and railway uses) were all dummy coded, which results in the total of seven dummies for each respondent. A person who had no cars in home and used railway, for example, will have two dummies of ‘car access Level 0’ and ‘railway used’ being set to 1, whereas the remaining five dummies are set to 0.

### Out-of-home activities

To measure the respondents’ out-of-home activities, we designed questions similarly to a travel diary. The respondents were asked to report all their trips made during the two survey days. Each chain of trips starts with departing from home, followed by successive (in terms of time) destinations, and finally a return home. A respondent can report multiple trip chains in each day. For each destination, the respondents were asked to select the performed activity from a given list of activities including work, school, business, shopping, daily leisure (e.g., trips for social purposes, eating, or entertainment), non-daily leisure (e.g., often trips involving longer distances than daily leisure trips, such as sightseeing or excursions), clinic/hospital visits, and pickups, and returning home. This activity list includes typical Japanese’s out-of-home activities and was designed by the authors of this study with referencing to the guideline in [29]. Then, the total number of times an activity is performed over two survey days was treated as a numeric variable showing the respondent’s frequency of performing that activity.

### Covariates

All variables other than motorized transport access and out-of-home activities were treated as covariates. The list of covariates includes: (1) gender (male = yes/no); (2) marital status (married = yes/no); (3) occupation status (company employee = yes/no; company manager or executive = yes/no; self-employed = yes/no; part-time job = yes/no; houseworker = yes/no; no employment (including retirees) = yes/no); (4) income levels (high/low income = yes/no corresponding to an annual income of 4 million JPY (approximately 37,432 USD at the time of writing) and above/below); (5) children status (living with children = yes/no); and (6) age (years, numeric). Additionally, 14 dummies were included for being in 14 regions out of the total 15 considered regions with the last region being treated as the reference category (The 15 considered regions are: Region 1: Kitahiroshima City, Hokkaido Prefecture; Region 2: Namie Town, Minamisoma City, Futaba Town, Fukushima Prefecture; Region 3: Hitachi City, Ibaraki Preferecture, Aizu area, Fukushima prefecture; Region 4: Machida City, Tokyo Capital; Region 5: Niigata City, Niigata Prefecture; Region 6: Shiojiri City, Nagano Prefecture; Region 7: Shizuoka City, Shizuoka Prefecture; Region 8: Kosai City, Shizuoka Prefecture; Region 9: Hamamatsu City, Shizuoka Prefecture; Region 10: Bisan District, Aichi Prefecture; Region 11: Eiheiji Town, Fukui Prefecture; Region 12: Yabu City, Hyogo Prefecture; Region 13: Shobara City, Hiroshima Prefecture; Region 14: Mitoyo City, Kagawa Prefecture; and Region 15: Tsukuba City, Ibaraki Prefecture).

### Statistical analysis

The descriptive statistics of the full sample include the frequencies of the binary variables and the means and standard deviations of the numeric variables. Where applicable, t-tests were conducted to assess the statistical significance of the difference between the two means of two compared groups over a continuous variable, and chi-squared tests were conducted to compare the frequencies of two groups over a binary variable. Prior to each t-test, a Levene’s test was conducted for assessing the statistical equality of variances of two compared groups for the variable in question. Based on the p-value derived from the Levene’s test, the succeeding t-test was conducted with the assumption of either unequal variances (for p-value of 0.05 or below) or equal variances (for p-value of above 0.05). The two compared groups were: (1) Male/female groups and; (2) Restricted (restricted life-space = yes) and unrestricted (restricted life-space = no) life-space groups. Both t-tests and chi-squared tests were conducted using the full sample.

The associations between motorized transport access, out-of-home activities, and composite LSA scores were assessed using generalized linear models (GLMs). GLMs were selected by observing the actual distribution of the composite LSA scores in the samples. For example, the observed composite LSA scores in the adjusted sample were all positive (ranging from 7 to 120) and statistically deviated from the normal distribution. For this reason, a gamma distribution was assumed, and the appropriate GLM was selected for this variable. In addition, we evaluated different models using male/female samples. The estimates provided by these models were used to derive conclusions concerning the associations separately for male/female groups. Conversely, the estimates provided by the models for the adjusted sample were used to draw joint conclusions concerning the associations for both males and females. In each sample, each predictor was tested using a univariate model, which included only the predictor and the dependent variable (i.e., the composite LSA scores), as well as a multivariate model that was adjusted for background factors represented by the aforementioned covariates. Only variables with coefficients that were significant in *both* univariate and multivariate models at the 90% confidence level were retained for interpretation.

SPSS for Windows (IBM SPSS Statistics for Windows, Version 27.0.1.0) was used for all significance tests and regression model estimations. Further, all GLMs were estimated with the Log link functions in SPSS.

## Results

The basic information of the respondents in the full sample is presented in Table 1. The full sample was also analyzed to determine the differences in the characteristics between males and females and older adults experiencing restricted/unrestricted life-space. The results of the regression models evaluated using male, female, and adjusted samples are presented in Table 2.

**Table 1:** Descriptive information of the full sample and compared samples

Characteristic	Full sample	Males	Females	Sig	Restricted life-space adults	Unrestricted life-space adults	Sig
Socio-demographic							
Mean age (SD)	70.63 (4.81)	70.73 (5.09)	70.34 (4.68)		72.09 (6.85)	70.48 (4.54)	***
Male	74.42%	100%	0%	N/A	67.80%	75.06%	*
Married	82.37%	85.18%	74.19%	***	81.36%	82.47%	
Having children & living together	59.49%	27.42%	28.45%		33.05%	27.16%	
Company employee	6.68%	7.86%	3.23%	***	3.39%	7.00%	
Company manager/executive	2.48%	3.23%	0.29%	***	2.54%	2.47%	
Self employed	6.98%	8.27%	3.23%	***	4.24%	7.24%	
Part-time job	12.23%	12.10%	12.61%		7.63%	12.67%	
Housework	13.73%	0.81%	51.32%	***	16.95%	13.42%	
No employment	53.41%	62.90%	25.81%	***	61.02%	52.67%	*
High income	14.78%	16.33%	10.26%	***	9.32%	15.31%	*
Car access							
Level 0: No cars in home	15.15%	11.79%	24.93%	***	22.03%	14.49%	**
Level 1: Only shared cars, NOT having a driver’s license	3.30%	2.22%	6.45%	***	12.71%	2.39%	***
Level 2: Only shared cars, HAVING a driver’s license	23.78%	22.28%	28.15%	**	22.88%	23.87%	
Level 3: Owning a car only	51.69%	56.05%	39.00%	***	38.98%	52.92%	***
Level 4: Both owning a car and sharing cars	6.08%	7.66%	1.47%	***	3.39%	6.34%	
Public transport used							
Bus	10.95%	10.48%	12.32%		9.32%	11.11%	
Railway	9.68%	10.58%	7.04%	*	5.08%	10.12%	*
Assistance in traveling							
Using supportive equipment	4.20%	4.44%	3.52%		45.76%	0.16%	***
With someone's help	3.23%	2.42%	5.57%	***	33.05%	0.33%	***
Out-of-home activities (activity number and SD)							
Work	0.23 (0.54)	0.27 (0.53)	0.13 (0.38)	***	0.11 (0.33)	0.24 (0.47)	***
Business	0.22 (0.85)	0.27 (0.89)	0.08 (0.46)	***	0.11 (0.61)	0.23 (0.7)	**
Shopping	2.3 (1.97)	2.26 (1.81)	2.45 (1.93)		2.07 (2.32)	2.33 (1.82)	
Daily leisure	0.47 (1.06)	0.51 (0.75)	0.34 (0.76)	***	0.39 (0.64)	0.47 (0.77)	
Non-daily leisure	0.2 (0.79)	0.24 (0.59)	0.07 (0.32)	***	0.09 (0.26)	0.21 (0.48)	**
Clinic/hospital	0.22 (0.56)	0.23 (0.48)	0.17 (0.51)	*	0.3 (0.79)	0.21 (0.45)	
Pickup	0.12 (0.46)	0.13 (0.59)	0.09 (0.37)		0.07 (0.57)	0.12 (0.47)	
Total	3.95 (2.93)	4.14 (2.45)	3.39 (2.31)	***	3.23 (3.07)	4.02 (2.3)	***
Life-space mobility indicators							
Composite life-space scores (SD)	77.41 (23.9)	78.8 (24.08)	73.35 (22.26)	***	39.09 (14.25)	81.13 (20.45)	***
Restricted life-space = yes	8.85%	8.06%	11.14%	*	100%	0%	N/A
Sample size	1,333	992	341		118	1215	

*Note.* Differences in the means (for continuous variables) and frequencies (for binary variables) were tested using t-tests and chi-squared tests, respectively

'***': *p*-value ≤ 0.01; '**': *p*-value ≤ 0.05; '*': *p*-value < 0.1

'N/A': not available; 'Sig.': significance; SD: standard deviation

**Table 2:** Estimation results for the composite life-space scores

Variable	Univariate estimate	95% CI	Multivariate estimate	95% CI
Male sample (*N* = 992)				
Car access				
Level 0: No cars in home	-0.15***	-0.21; -0.08	-0.13***	-0.2; -0.06
Level 1: Only shared cars, no driver’s license	-0.19**	-0.34; -0.05	-0.14*	-0.28; 0
Level 3: Owning a car only	0.09***	0.05; 0.13	0.08***	0.04; 0.12
Public transport used				
Railway	0.09**	0.02; 0.16	0.08**	0.01; 0.15
Out-of-home activities				
Work	0.13***	0.1; 0.17	0.07***	0.02; 0.12
Business	0.06***	0.03; 0.08	0.03**	0.01; 0.06
Shopping	0.02***	0.01; 0.03	0.02***	0.01; 0.03
Daily leisure	0.03***	0.01; 0.05	0.04***	0.02; 0.05
Non-daily leisure	0.05***	0.02; 0.07	0.04***	0.02; 0.06
Hospital/clinic	-0.05**	-0.08; -0.01	-0.04*	-0.07; 0
Pickup	0.07***	0.03; 0.11	0.08***	0.04; 0.12
Socio-demographic characteristics				
Region 5	-0.06*	-0.11; 0	-0.06*	-0.11; 0
Region 10	0.07*	0; 0.14	0.07*	0; 0.14
Company employee	0.2***	0.12; 0.28	0.2***	0.12; 0.28
Company manager	0.11*	-0.01; 0.23	0.11*	-0.01; 0.23
Part-time job	0.12***	0.06; 0.18	0.12***	0.06; 0.18
No employment	-0.16***	-0.2; -0.11	-0.16***	-0.2; -0.11
High income	0.11***	0.05; 0.16	0.11***	0.05; 0.16
Age	-0.01**	-0.01; 0	-0.01**	-0.01; 0
Female sample (*N* = 341)				
Car access				
Level 1: Only shared cars, no driver’s license	-0.17**	-0.32; -0.03	-0.13*	-0.27; 0.02
Level 3: Owning a car only	0.12***	0.05; 0.19	0.11**	0.03; 0.18
Out-of-home activities				
Shopping	0.03***	0.01; 0.05	0.03***	0.01; 0.05
Daily leisure	0.08***	0.04; 0.13	0.07***	0.02; 0.12
Pickup	0.11**	0.01; 0.2	0.12**	0.02; 0.21
Socio-demographic characteristics				
Region 12	-1.08***	-1.73; -0.43	-1.06***	-1.71; -0.41
No employment	-0.08**	-0.17; 0	-0.28**	-0.48; -0.08
Adjusted sample (*N* = 610)				
Car access				
Level 0: No cars in home	-0.09**	-0.15; -0.02	-0.07*	-0.14; 0
Level 1: Only shared cars, no driver’s license	-0.16**	-0.29; -0.04	-0.1*	-0.23; 0.02
Level 2: Only shared cars, with driver’s license	-0.07**	-0.13; -0.01	-0.05*	-0.12; 0.01
Level 3: Owning a car only	0.13***	0.08; 0.18	0.1***	0.05; 0.16
Public transport used				
Railway	0.1*	0; 0.2	0.09*	-0.01; 0.19
Out-of-home activities				
Work	0.16***	0.11; 0.22	0.1**	0.03; 0.18
Business	0.07***	0.03; 0.11	0.04*	0; 0.08
Shopping	0.03***	0.02; 0.04	0.04***	0.02; 0.05
Daily leisure	0.07***	0.04; 0.11	0.06***	0.03; 0.09
Non-daily leisure	0.09***	0.03; 0.15	0.06**	0; 0.12
Hospital/clinic	-0.08***	-0.13; -0.02	-0.06**	-0.11; -0.01
Pickup	0.08***	0.02; 0.14	0.08***	0.02; 0.13
Socio-demographic characteristics				
Region 12	-0.84***	-1.3; -0.38	-0.8***	-1.26; -0.34
Male	0.08***	0.03; 0.13	0.08**	0.01; 0.16
Houseworker	-0.07**	-0.13; -0.01	-0.15**	-0.29; -0.02
No employment	-0.08***	-0.13; -0.03	-0.21***	-0.34; -0.09

*Note.* The multivariate models were adjusted for all covariates such as gender, marital status, and regional characteristics. Only parameters significant in both univariate and multivariate models are presented. The parameters in this table are raw parameters reported by SPSS with Log link functions. Consequently, the natural exponentials of these parameter give the effects of the independent variables on the composite LSA scores. Negative parameters indicate associations between independent variables and reductions in the composite LSA scores, and positive parameters indicate associations with increases in the composite LSA scores

'***': *p*-value ≤ 0.01; '**': *p*-value ≤ 0.05; '*': *p*-value < 0.1; 'No.': number; 'CI': Confidence Intervals

As mentioned, males dominated the full sample whereas the adjusted sample was more gender balanced and reflective of the gender distribution of the population. More than half of the respondents lived with their children and were without an employment. Only about 15% of the respondents had incomes of more than 4 million JPY (approximately 37,432 USD at the time of writing) and, therefore, were considered as high-income adults. The sample showed a high level of car access, with nearly 85% of older adults having accesses to either shared cars or their own cars. Consequently, less than 5% of the respondents had to depend on travel equipment/human assistance. Regarding out-of-home activities, each respondent conducted nearly four activities on average during the two survey days, and shopping accounted for more than half of their activities. The second most common activity was daily leisure activity, which was conducted 0.47 times per two days (approximately one activity per four days). Finally, the average composite LSA score of all respondents was 77.41, which is higher than that of a sample used in another study in Japan [23] at 56.2. The participants in [23] were older adults who had joined the Japanese long-term care insurance system and had to visit daycare centers. This fact might indicate their poorer health, which can limit their life-spaces.

Comparisons between male/female and life-space restricted/unrestricted groups provided additional information regarding the full sample. The percentages of males with Level 3 & Level 4 of car access (50.65% and 7.66%) were statistically higher than that of their female counterparts (39% and 1.47%), and this was in line with the corresponding comparison on the of Level 0 & Level 1 of car access. A similar pattern was observed in comparisons between the life-space restricted & unrestricted groups over the car-access indicator, where 12.71% and 38.98% of the life-space restricted individuals had Level 1 & Level 3 of car access, respectively, and the corresponding numbers of their counterparts were 2.39% and 52.92%, respectively. For the public-transport-used indicator, the difference was observed only for railway use where the male and life-space unrestricted groups showed higher usages than their counterparts. For out-of-home activities, male and life-space unrestricted respondents conducted 0.27 and 0.24 work activities per two days, respectively, which are statistically higher than those of the counterparts at 0.13 and 0.11 activities, respectively. As expected, the mean of the composite LSA scores of the male group (78.8) was statistically higher than that of the female group (73.35), which trend has also been reported in previous studies [3, 16].

In the *male sample*, we found that car accesses of Level 0 & 1 were associated with decreases in the composite LSA scores, as evidenced by the significant and negative coefficients. Conversely, car access of Level 3 and only railway used were associated with increases in the composite LSA scores. On the other hand, all out-of-home activities were found to be associated with increased composite LSA scores, except for hospital/clinic with decreased composite LSA scores. Finally, only a few covariates were found to be associated with the composite LSA scores. For example, those without an employment were associated with reduced composite LSA scores, whereas those who had high incomes were associated positively.

In the *female sample*, we observed only a few significant associations between motorized transport access and the composite LSA scores. Only car accesses of Level 1 & Level 3 were associated with decreased and increased composite LSA scores, respectively. Several significant associations could also be observed for the variables of out-of-home activities; for example, only shopping, daily leisure, and pickup activities were found to be associated with increases in composite LSA scores. The associations of the remaining socio-demographic characteristic variables mostly became insignificant.

When males and females were pooled in the *adjusted sample*, the pattern of associations changed slightly. First, all variables with significant coefficients in both the male and female samples (i.e., car accesses of Level 1 & Level 3, shopping/daily leisure/pickup activities, and no employment) were again observed with significant coefficients in the adjusted sample. Next, considering the variables that were significant in either the male or female samples, some were still significant in the adjusted sample (including car access of Level 0; railway used; work, business, non-daily leisure, and hospital/clinic activities; and being in region 12), whereas several became insignificant (e.g., being in regions 5 & 10). Particularly, being houseworker was the only socio-demographic variable that was found being significant only in the adjusted sample. Examining the magnitudes of the coefficients for car accesses of Level 1 & Level 2 revealed that although depending on shared cars was associated with reduced composite LSA scores, the reductions were greater for those who do not have a driver’s license.

In all samples, the coefficients for car access of Level 4 (i.e., both owning a car and sharing cars) were insignificant (and, hence, being not reported here). This implies that additional access to shared cars might not add any value to those who already have their own cars. Among the socio-demographic variables, no employment and being in region 12 (Yabu city, Hyogo prefecture) were strong correlates of LSM, all with negative coefficients. The negative coefficient of being in region 12 was in some sense indicative of the characteristics of the corresponding region, as this city is almost a mountainous region with a limited public transport system.

## Discussion

This study explored motorized transport access and out-of-home activities as correlates of LSM, which is a common concept used to study the mobility of older adults. From the results obtained using different models and different samples, it was found that *the different levels of motorized transport access (car, bus, and railway) and presence of different performed out-of-home activities were associated with different composite LSA scores*. We therefore highly suggest considering these factors in efforts to improve LSM of older adults.

The results shown in Table 2 confirm our hypothesis H1, as it was found that different levels of motorized transport access (car and railway) were associated differently with composite LSA scores. An interesting fact was that car accesses of levels lower than Level 2 (i.e., having a driver’s license and sharing a car only) were all associated with decreased composite LSA scores, whereas only car access of Level 3 (i.e., owning a car only) was associated with increased composite LSA scores. Therefore, *access to shared cars only can still be an indicator of a restricted LSM*. The results from models with the male, female, and adjusted samples revealed a general trend in the associations between motorized transport access and LSM, that is *higher levels of access to transport modes (i.e., owning a car or using railway), in contrast to lower levels (i.e., no cars in home or only shared cars), were positively associated with composite LSA scores*. This finding has signified the role of motorized transport access, and car access in particular, in the LSM of older adults from that several considerations of interest for policymakers can be derived. Specifically, although the availabilities of certain travel modes differ between regions in a country and between countries, the results of our study indicated that generally higher levels of motorized transport access can lead to improved LSM. Therefore, to improve older adults’ long-distance LSM, the provision of transportation alternatives (particularly car use) is important. Offering more transportation options was also recognized in previous studies as a key factor for improving the mobility of older adults [24–26]. However, it is not always feasible to invest in developing new transport facilities due to economic reasons, particularly in rural and remote areas where travel demands are generally low and cannot attract investors, which situation has been observed in Japan [27]. Consequently, older adults living in these areas are more mobility-disadvantaged than those living in well-developed areas. In this case, we suggest that Mobility-as-a-Service (MaaS) applications can provide alternative transportation options at lower costs, thereby being more practically feasible for implementation. This suggestion agrees with that provided in [26] wherein the authors highlighted the role of a range of mobility services in improving the mobility of older adults, including subsidized taxi, door-to-door transport services, and new forms of demand services. Such applications can therefore be a potential strategy for improving LSM for older adults living in rural and remote areas, particularly when the use of public transport can cause some difficulties for such adults [24]. At the time of writing, a special form of MaaS called ‘On-demand taxi service’ was being experimented in the Smart Mobility Challenge project in Japan. Shared uses and fixed get-on/get-off locations are two characteristics of the on-demand taxi service, which enable traveling with lower costs (e.g., compared to normal taxis) while remain acceptable flexibility (e.g., compared to fixed-schedule buses). The on-demand taxi service was expected to be a financially viable solution for improving transport access.

The second hypothesis H2 was also confirmed by the results in Table 2, as different out-of-home activities were differently associated with the composite LSA scores. This result suggests two considerations for policymakers that are interested in improving the LSM of older adults. First, the estimated parameters revealed that older adults who had more work, business, shopping, daily leisure, non-daily leisure, and pickup activities were associated with increased LSM scores. Conversely, those who visited the clinic or hospital more frequently were associated with decreased LSM scores. This result confirms the finding in [8] that fewer work and business needs with age can be a cause of restricted mobility in older adults. In addition, this finding suggests that *older adults who go to clinics/hospitals more frequently, or those who have less and/or reduced shopping and daily leisure activities, are likely to suffer from reduced LSM*. Policy interventions for this reduced-LSM cohort, such as free travel tickets, can lead to their improved LSM. Another suggestion from this result is that *if the needs for specific activities can be triggered, the resultant LSM will be improved for older adults*. Triggering the needs for shopping and daily leisure activities are two potential directions for policy intervention (although the work activity had the strongest association with LSM, this factor appears to not be suitable for policy interventions). This result agrees with that presented in [28] where “active in daily life” (who had a goal of “going outside everyday”) was found to be a strong predictor of LSM. In this study context, we suggest a simple intervention via collaboration between transportation and other relevant industries. Considering shopping and eating activities, small-scale retailers and restaurants can cooperate with transport operators to provide incentives such as a free taxi or free shuttle bus service for going shopping at designated stores or eating at designated restaurants. This type of intervention, if successfully implemented, can yield multiple outcomes for all stakeholders while achieving its primary goal of improving the LSM of older adults. Although online shopping and food delivery can directly affect this form of intervention, such trends require users to be technologically savvy for efficient use, which may be a barrier for some older adults. Moreover, not all goods are suitable for delivery.

Although motorized transport access and out-of-home activities were investigated as separate variables in our models, we do not exclude the possibility of a more complex relationship between these variables, which may influence LSM. In future studies, the influence of motorized transport access as an enabler for out-of-home activities should be investigated. For older adults, particularly those living in areas with insufficient public transport systems such as rural and remote areas, the limited travel mode options may result in a refrain from traveling if the specific out-of-home activities are not urgent. Therefore, the provision of evidence for this hypothesis may contribute to increasing the LSM of older adults.

A point must be noted regarding the generalizability of the results of this study. The results and our suggestions in this study are valid only to areas with low accessibilities to daily services, which are typically rural and distant areas where these services are sparsely distributed. In these areas, improved mobility through providing more motorized transport modes potentially leads to improved LSM, which ultimately can contribute to overall health and well-being of the residents. For other well-serviced areas, the need to travel over long distances might be eliminated and, hence, making the implications from this study irrelevant.

Finally, this study has several limitations that we hope will be overcome in future studies. First, we needed to randomize the full sample to create a more gender-balanced sample that can better represent the population. Although this randomization does not affect the estimates of the models for males and females, we believe that it would be ideal if a stratified sampling technique could be applied to yield a more gender-balanced sample. Another limitation was that public transport access was not measured accurately due to the privacy restrictions in the survey. In addition, the number of motorized modes was limited because of the existing availabilities of these modes in the regions involved in our study. When more motorized modes are investigated, their effects on LSM may further affect social policy interventions. We suggest that the framework used in this study be applied to regions in which new and emerging travel modes are available, such as MaaS applications, to further improve the LSM of older adults.

## Conclusions

This study considered the associations between motorized transport access, out-of-home activities, and LSM, which have not yet been fully investigated in clinical and aging studies. Using data from older adults aged 65 and above in 15 experimental regions in Japan, we tested various regression models to examine the potential associations. Overall, we found that motorized transport access, including owning a car and using railway, as well as work, business, shopping, daily leisure, non-daily leisure, and pickup activities, were associated with larger composite LSA scores, thereby resulting in increased LSM scores. Conversely, lower levels of car access, such as no cars in home or only sharing a car, more clinic/hospital activities, and no employment are factors that imply a restricted LSM.

Based on these findings, we suggest that policymakers should improve transport access for older adults, such as introducing MaaS as an alternative to car access. More attention should be paid to the LSM of older adults with high clinics/hospital activities. Out-of-home activities may also be triggered by collaborations between shopping retailers and transportation operators, thereby improving the LSM of older adults.

## Data Availability

The data that support the findings of this study are available from the METI, but restrictions apply to the availability of these data, which were used under license for the current study; therefore, the data is not publicly available. However, the data is available from the authors upon reasonable request and with permission from the METI. General information on the data can be found on the METI’s website for the FY2020 Smart Mobility Challenge Project: https://www.meti.go.jp/english/press/2021/0402_002.html.

## Abbreviations

COVID-19: Coronavirus disease 2019
LSA: Life-Space Assessment
LSM: Life-space mobility
GLM: Generalized linear model
MaaS: Mobility-as-a-Service
METI: Ministry of Economy, Trade, and Industry, Japan
